# Does the settling column method underestimate phytoplankton sinking speeds?

**DOI:** 10.1098/rsos.231455

**Published:** 2024-02-07

**Authors:** Kevin T. Du Clos, Brad J. Gemmell

**Affiliations:** Department of Integrative Biology, University of South Florida, 4202 East Fowler Avenue, Tampa, FL 33620, USA

**Keywords:** diatom, phytoplankton, methods, sinking, settling column method, SETCOL‌

## Abstract

Phytoplankton sinking is a major component of vertical ocean carbon and nutrient fluxes, and sinking is an integral component of phytoplankton biology and ecology. Much of our understanding of phytoplankton sinking derives from the settling column method (SETCOL) in which sinking speeds are calculated from the proportion of cells reaching the bottom of a water-filled column after a set time. Video-based methods are a recent alternative to SETCOL in which sinking speeds are measured by tracking the movement of individual cells in a salinity-stratified water column. In this study, we present the results of a meta-analysis showing that SETCOL produces significantly and consistently lower sinking speeds than the video method. Next, we perform a particle image velocimetry analysis, which shows that the observed discrepancy in sinking speeds between the two methods can probably be explained by weak convection currents in the SETCOLs. Finally, we discuss the implications of these results for the interpretation of past and future phytoplankton sinking speed measurements and models that rely on those measurements.

## 1. Introduction

Phytoplankton sinking is a major contributor of vertical nutrient and carbon fluxes from surface water to depth [1]. Accurate estimates of phytoplankton sinking speeds are, therefore, crucial for understanding global carbon and nutrient cycling as well as phytoplankton population dynamics. Phytoplankton sinking speeds are most commonly quantified using settling column (SETCOL)-based methods. These methods were formalized by Bienfang in the 1980s as SETCOL [2] but have been in use since the 1910s [3] and are still widely used, e.g. [4–7]. In SETCOL, a cylindrical column is filled with water with a homogeneous suspension of phytoplankton. After a set time interval, the water at the bottom of the column and the overlying water are collected and the phytoplankton in each fraction quantified by weight, cell counts, fluorescence, etc. Sinking speed is calculated as the proportion of the total phytoplankton in the bottom volume times the length of the column divided by the elapsed time [2]. A water jacket is used to minimize convection currents. SETCOL is inexpensive to perform and has provided important details on how mean sinking speed varies across phytoplankton taxa [8] and how diatom sinking is affected by temperature, salinity, nutrient depletion, irradiance and reproductive status [8–13]. SETCOL-derived sinking speeds have also been incorporated into ecosystem and nutrient-cycling models, e.g. [14–19].

Recently, video methods capable of tracking sinking behaviour of individual cells have been employed in a limited number of studies [20–24]. In these methods, phytoplankton are introduced to the top of a large filming vessel filled with several litres of salinity stratified water, and their sinking is recorded with video microscopy as they pass through the field of view, near the centre of the filming vessel and far from any walls. Individual sinking cells are then tracked to quantify sinking speeds. The use of a large volume of salinity stratified water suppresses convection during filming, as demonstrated by dye studies (see fig. S1 in [24]). Results from studies based on video methods have shown that sinking speed varies not just between populations and cultures but also between individual cells, with cells from the same culture exhibiting a broad range of sinking speeds. They have also provided evidence of unsteady sinking behaviours in large diatoms on timescales of seconds [22,23] and of strong and rapid (within 1 h) increases in mean sinking speed when cells cross a nutricline into high-nutrient conditions [24].

Past studies have noted a discrepancy between video and SETCOL methods [20,21] but have not systematically investigated this discrepancy. Given the importance of phytoplankton sinking speeds in ocean modelling, it is important to determine why such a discrepancy exists. We performed a meta-analysis of sinking speeds for a diatom genus that has been investigated using both methods in order to identify the magnitude of the discrepancy. We then performed particle image velocimetry (PIV) experiments with a modified SETCOL to determine the underlying fluid mechanics in SETCOLs and to determine if convection driven currents with magnitudes similar to phytoplankton sinking speeds exist in the columns.

## 2. Meta-analysis

We collected sinking speed data for nutrient replete *Coscinodiscus* spp. diatoms from the literature to compare sinking speeds (*V*) derived from the SETCOL and video methods. *Coscinodiscus* was chosen owing to the availability of sinking speed data from both methods. We found five articles reporting SETCOL data [9,11,25–27], three reporting video-based data [21,22,24] and one reporting both [20] (table 1). When minimum and maximum values were reported for cell diameter (*d*), the median was used for analyses. One far outlier data point from reference [21] was excluded from analysis. We also included unpublished video method data for *Coscinodiscus granii* with a diameter of 60.3 μm. Analyses were performed in R v. 4.3.1 [28] using the AICcmodavg, glmtoolbox and interactions packages [29–31]. Results were plotted with Matplotlib [32] in Python 3.10 (python.org).

**Table 1.** Sinking speeds (*V*) and cell diameters (*d*) for *Coscinodiscus* species derived from SETCOL and video-based methods. (The asterisk (*) refers to unpublished data.)

species	mean *d*	min. *d*	max. *d*	mean *V*	mean *V*	method	reference
	(μm)	(μm)	(μm)	(mm s^−1^)	(m d^−1^)		
*C. wailesii*	45.7			0.0028	0.24	SETCOL	[9]
*C. wailesii*	90.4			0.0126	1.09	SETCOL	[26]
*C. concinnus*	201			0.0111	0.96	SETCOL	[11]
*C. wailesii*		160	350	0.0207	1.79	SETCOL	[27]
*C. wailesii*		160	350	0.0245	2.12	SETCOL	[27]
*Coscinodiscus* sp.		200	500	0.022	1.9	SETCOL	[20]
*Coscinodiscus* sp.		200	500	0.0713	6.16	video	[20]
*C. wailesii*	216			0.0475	4.1	video	[21]
*C. radiatus*	56			0.0486	4.2	video	[21]
*C. radiatus*	34			0.0451	3.9	video	[21]
*C. wailesii*	102			0.029	2.51	video	[24]
*C. wailesii*	300			0.052	4.49	video	[22]
*C. granii*	60.3			0.024	2.07	video	*

**Figure 1.**
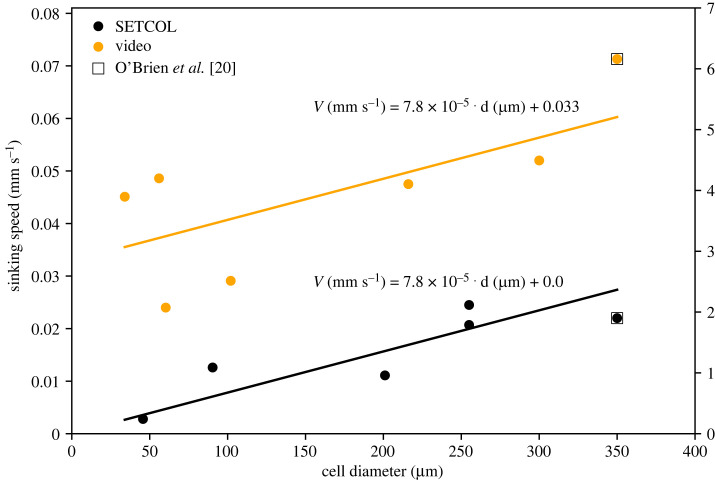
Sinking speeds for *Coscinodiscus* spp. under nutrient replete conditions from published SETCOL [9,11,20,26] and video data ([20,22,24], and unpublished data for *C. granii*). O’Brien *et al.* [20] compared video and SETCOL methods for the same population of cells (open squares). The lines and equations represent the results of a generalized linear model of (*V* in mm s^−1^) versus cell diameter (*d* in μm) and method (*R*^2^ = 0.80).

Analysis of sinking speeds from the literature revealed that SETCOL produced consistently and significantly lower sinking speeds than the video method (figure 1). Results of Welch’s *t*-tests (*α* = 0.05) showed that sinking speeds were significantly lower for SETCOL than for the video method (*p* = 0.0016), whereas cell diameters did not differ significantly (*p* = 0.57).

To explicitly include the effects of cell diameter on sinking speed, generalized linear models (GLM) were run with sinking speed as the dependent variable and cell diameter and sinking speed method as independent variables. An initial GLM showed no significant interaction between cell diameter and method (*p* = 0.59), and the Akaike information criterion value was lower for a reduced model without interactions, so we based our analyses on the reduced model. The GLM showed significant effects of cell diameter (*p* = 0.005) and method (*p* < 0.0001) on sinking speed (figure 1, adjusted *R*^2^ = 0.80). Based on the model, sinking speeds from SETCOL were approximately 0.033 mm s^−1^ (2.85 m d^−1^) slower than those from video methods, independent of cell diameter.

## 3. Particle image velocimetry

To test for convection currents that could affect SETCOL measurements, we built a SETCOL with dimensions based on standard SETCOL (2.3 cm inner width, 50 cm high, 265 ml volume) as described by Bienfang [2] but with a square cross-section, which was necessary to provide optical access and prevent optical distortion to record videos for PIV analysis. The column was filled with water seeded with hollow glass spheres (diameter = 9−13 μm, *ρ* = 1.10 ± 0.05 g cm^−3^; LaVision). As in SETCOL [2], the column was placed in a water jacket ($25{\;}^{\circ }C$) to minimize convection and capped to prevent airflow in the room and evaporation from generating minute surface currents. Videos were recorded at seven vertical slices through the centre of the tank. These videos were used to calculate velocity fields, which were then interpolated to reconstruct a three-dimensional, two-component flow field.

**Figure 2.**
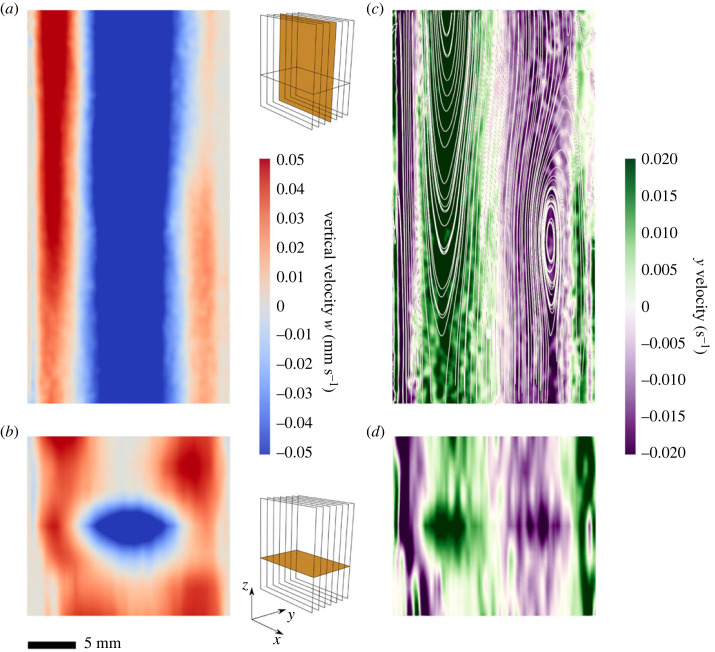
A particle image velocimetry (PIV) experiment with a modified SETCOL shows convection currents. (*a*,*b*) Front and side views of the vertical velocity component *w*, where a positive vertical velocity is upwards. (*c*,*d*) Front and side views of the *y*-component of vorticity. Insets indicate the locations of the displayed views. Grey streamlines are shown in (*c*).

Videos were recorded with an Edgertronic SC1 camera (Sanstreak Corp.) at 10 frames s^−1^ with illumination from a 532 nm, 600 mW continuous wave diode pumped solid state laser spread into an approximate 1 mm thick light sheet parallel with the front face of the column. At the vertical centre of the tank, seven evenly spaced slices were recorded from front to back (figure 2; electronic supplementary material, figures S2 and S3). To test for variations in the flow with height, one additional slice was recorded 20 cm from the bottom of the tank, centred between the front and back of the tank (electronic supplementary material, figure S2). One 7 min video was recorded for each view. The field of view was approximately 36 × 45 mm.

Velocity vectors were calculated using PIV processing in DaVis 8 (LaVision) with decreasing interrogation window sizes—one pass at 32 × 32 px followed by three passes at 24 × 24 px—with no post processing. A frame increment of 10 was used for processing (d*t* = 1 s). A mean velocity field was calculated for each video by time averaging velocities. Mean velocity fields from the seven views at the same depth were combined by linear interpolation to produce a three-dimensional, two-component representation of the flow in the column. Mean velocities are reported with standard deviations based on the seven views (mean ± s.d.).

PIV analysis of the simulated SETCOL in a water jacket revealed persistent convection currents (figure 2; electronic supplementary material, figures S2 and S3). The flow was toroidal with upwelling currents near the walls and a downwelling core. Vertical velocities in the test section were upwards overall (mean: 0.012 ± 0.017 mm s^−1^; maximum: 0.054 ± 0.008 mm s^−1^). Mean horizontal velocities were less than 0.001 mm s^−1^. The large, salinity stabilized water column used in video-based measurements has been shown to exhibit negligible mixing over the course of an experiment (see fig. S1 in [24]).

## 4. Discussion

Obtaining accurate phytoplankton sinking speeds is important as these speeds can be used in estimations of nutrient and biomass fluxes in the ocean, e.g. [14–19]. The most widely used method of obtaining sinking speeds of phytoplankton for the last several decades has been the SETCOL method. Past studies have noted differences between SETCOL and video-based sinking speed measurements [20,21]. Our meta-analysis supports these findings and demonstrates that SETCOL produces sinking speeds consistently 0.03 mm s^−1^ (2.6 m d^−1^) slower than video methods (figure 1). Owing to the lack of a significant interaction between cell diameter and method, slopes of sinking speed versus cell size are consistent between video and SETCOL methods with only the intercepts differing. The lack of interaction suggests that physical differences owing to cell size or shape do not impact the observed methodological discrepancy. The fact that the discrepancy between methods is consistent with cell size means that small, slower sinking cells or particles are more strongly affected relative to their speeds; for 46 μm diameter and smaller cells, the discrepancy is more than an order of magnitude.

To investigate the mechanism for the sinking rate discrepancy between the two methods, we performed a PIV experiment in which we visualized the fluid velocities within a modified SETCOL. Previous work has demonstrated minimal convective currents within the large, salinity stabilized columns used for the video method [24]. Our results show that weak but persistent convection currents persist in SETCOL columns, even when immersed in temperature-controlled water baths (figure 2). These convection currents, while slow, are within the range of diatom sinking speeds and more than an order of magnitude greater than small cell (≤46 μm diameter) sinking speeds, with maximum observed vertical flow speeds of 0.05 mm s^−1^, and could thus lead to substantial underestimates of phytoplankton sinking speeds. The flows observed in the SETCOL column are also of similar magnitude to the sinking speed discrepancy observed in the meta-analysis, with consistent upwards flow near the column walls and downwelling flows in the centre of the column (figure 2). It has been established that convective flows can slow the bulk sinking rate of particles [33–35]. Cells or particles at the centre of the column temporarily sink faster than their intrinsic sinking rate owing to the downwelling flow, but as this flow approaches the bottom, it spreads and a portion of the cells are advected towards the column walls where they are carried upwards by the return flow. The process can repeat several times, thereby slowing the average time it takes for a population of cells or particles to reach the bottom. Additionally, wall effects [36] and hindered settling [37], both of which slow particle sinking, are stronger in the narrow vessels used in the SETCOL method. By contrast, the video method minimizes convection currents, wall effects and hindered settling by using a salinity-stratified water column and measuring sinking speeds of cells far from vessel walls and at low volume fractions.

We were limited in the availability of sinking speed data, particularly for the video method. To minimize sources of variance, we focused our meta-analysis on *Coscinodiscus*, a genus of large centric diatoms, but there were variations in methodology and culture conditions even within this limited dataset. The consistency of the observed trends despite these limitations and a direct comparison by O’Brien *et al.* [20], who used both methods to measure sinking speeds for the same *Coscinodiscus* population and reported 0.050 mm s^−1^ (4.3 m d^−1^) faster sinking speeds for video-based measurements versus SETCOL [20], both suggest that the observed discrepancy between the methods is robust. The identification of convection currents as a physical mechanism suggests that this discrepancy should hold for cells and particles of all types, but additional direct comparisons of the methods (as in [20]) should be performed to confirm this, particularly for cells smaller than those included in our meta-analysis.

SETCOL is well established and inexpensive to perform and in some cases may be better suited than video-based methods to a particular study, for example, when measuring sinking speeds for many samples at once or when quantifying sinking speeds for different biomass fraction, such as particle organic carbon, particulate organic nitrogen and chlorophyll, separately as in [38], so it will probably remain a popular method. We therefore offer suggestions to minimize the sources of biases for future SETCOL measurements. First, the diameter of SETCOLs should be increased to minimize biases from convection currents, wall effects and hindered settling. Second, we suggest using a salinity stratified SETCOL when possible, as salinity stratification is an effective method for preventing vertical convection. Third, when reporting SETCOL data, details of the method used, such as the dimensions of the column, should be provided to facilitate comparisons between studies. Finally, we suggest using video-based methods in conjunction with SETCOL to verify sinking speeds and correct biases as needed. Sinking speed biases are likely to differ between experimental set-ups, and calibrating a system to results from video measurements will improve reproducibility and facilitate comparisons between studies. Systematic studies combining SETCOL and video-based methods could be performed to examine the effects of SETCOL diameter, salinity stratification, etc., on SETCOL derived sinking speeds.

## Data Availability

Data and metadata are available through the figshare repository https://doi.org/10.6084/m9.figshare.24059154 [39]. Electronic supplementary material is available online [40].
